# Exploring the association between the proximity to and density around schools of retailers selling IQOS products and youth use of heated tobacco products: evidence from the 2020-2021 COMPASS study

**DOI:** 10.24095/hpcdp.44.1.01

**Published:** 2024-01

**Authors:** Hunter Mott, Scott T. Leatherdale, Adam G. Cole

**Affiliations:** 1 Faculty of Health Sciences, Ontario Tech University, Oshawa, Ontario, Canada; 2 School of Rehabilitation Sciences, Queen’s University, Kingston, Ontario, Canada; 3 School of Public Health Sciences, University of Waterloo, Waterloo, Ontario, Canada

**Keywords:** heated tobacco product, HTP, heat-not-burn product, tobacco, retailer density, retailer proximity, adolescent, IQOS

## Abstract

**Introduction:**

Heated tobacco products (HTPs) are novel tobacco products that may appeal to youth. This study explored whether HTP retailer proximity and density to secondary schools were associated with youth use of HTPs in four Canadian provinces.

**Methods:**

An online search between November 2020 and March 2021 identified retailers selling IQOS devices and HEETS (tobacco sticks used in IQOS) within 500m, 1000m and 1500m radius circular buffer zones around high schools (N=120) participating in the COMPASS study in 2020–2021. Retailer proximity/density data were linked to cross-sectional student-level data (N=40636 students), and multilevel regression models examined the association between HTP retailer proximity and density and current HTP use, controlling for relevant covariates.

**Results:**

While only 10.0% of schools had at least one retailer selling IQOS devices within 1000m of the school, 65.0% of schools had at least one retailer selling HEETS. The school a student attended accounted for 23.7% of the variability in the likelihood of currently using an HTP. However, HTP retailer proximity to and density around schools were not significantly associated with the likelihood of students currently using HTPs.

**Conclusion:**

While the school a student attended accounted for a significant amount of variability in HTP use, these findings suggest that students may be obtaining HTPs through other, non-retail sources. Continued monitoring is warranted as HTP use among youth may change.

**Highlights**

- This study is the first to explore the association between the proximity to and density of IQOS retailers to secondary schools and students’ use of heated tobacco products (HTPs).
- The school a student attended significantly affected the likelihood that they currently use HTPs.
- It is necessary to continue monitoring HTP retailer proximity to and density near schools and prevalence of HTP use among youth as HTPs become more common in Canada.

## Introduction

Heated tobacco products (HTPs, or “heat-not-burn products”) heat tobacco sticks into an aerosol that the user inhales.^1^,^2^ HTPs differ from traditional cigarettes, which are burnt so that the user inhales tobacco smoke, and from e-cigarettes, which heat a nicotine-containing solution into an aerosol that the user inhales.^1^ IQOS is a common brand of HTP,^2^ whereas HEETS are sticks of tobacco that are used with IQOS devices. Both can be found in specialty stores (such as IQOS’ Q-lab stores and boutiques) and non-specific stores (e.g. gas stations and convenience stores).^3^ IQOS was introduced to the Canadian market in 2017^4^ and is regulated under the *Tobacco and Vaping Products Act*.^5^

Since HTPs heat rather than burn tobacco, levels of carcinogens and toxicants produced are lower than those emitted by cigarettes,^6^ which contributed to the decision made by USA to approve IQOS as a “modified risk tobacco product.”^7^ However, an experimental study showed human bronchial cell cytotoxicity levels to be lower when using HTPs compared to when smoking cigarettes, but higher than during e-cigarette use.^2^ Furthermore, a systematic review suggests a positive correlation between HTP use and the incidence of respiratory complications, including airway remodelling and inflammation.^8^ Given the novelty of HTPs, research on their long-term health effects is limited.^8^

Although IQOS products were proposed as a substitute to help reduce smoking behaviours in current cigarette smokers, adolescents may use these products instead of cigarettes.^1^,^9^-^12^ While US data indicate that awareness of HTPs among youth increased between 2017 and 2020,^1^,^9^ ever and current use has remained low (<3%).^9^-^11^ Data from the Republic of Korea also indicate that ever use of HTPs remained low (2.9%) one year after their introduction onto the market in 2017.^12^ Despite a low prevalence of use, 33.0% of youth in Canada and 40.9% of youth in the USA reported being interested in trying IQOS in 2017, and 40.1% of youth in Canada and 46.1% of youth in the USA were susceptible to trying IQOS in the future.^1^

The diffusion of innovation theory proposes a mechanism for adoption and increased prevalence of a new idea, product or behaviour, for example, the use of HTPs, over time; youth who use HTPs in the early stages, that is “innovators” or “early adopters,” may influence others to try the product.^13^ As more individuals try the product, its diffusion in the population grows.

Evidence indicates that students in schools with a higher concentration of tobacco retailers nearby are more likely to smoke cigarettes.^14^ Tobacco product retailers near secondary schools may influence adolescent smoking behaviours by offering adolescents opportunities to conveniently access products and notice tobacco product marketing strategies.^14^ According to a cohort study in the UK, adolescents exposed to point-of-sale displays involving tobacco products were more susceptible to smoking.^15^ Given this evidence for an association between exposure to tobacco product marketing and risk of future tobacco use, investigating the possible association between the density and proximity of IQOS retailers and secondary schools is warranted.

To our knowledge, only one study, conducted in Israel, has examined the density of and proximity to schools of IQOS retailers; the authors reported an average of 1.60 retailers within a 400m radius of schools and an average of 8.73 retailers within a 1000m radius.^16^ We are not aware of any published studies that evaluate the association between IQOS retailer density and proximity to secondary schools and youth use of HTPs. 

The objectives of our study were to examine whether (1) IQOS retailer proximity to schools and (2) IQOS retailer density near schools were associated with past 30-day (current) HTP use in a convenience sample of Canadian students.

## Methods

***Sample selection***

This study used data from the 2020–2021 Cannabis, Obesity, Mental health, Physical activity, Alcohol, Smoking, and Sedentary behaviour (COMPASS) study,^17^ which included 53469 students in Grades 9 through 12 (secondary I–V in Quebec) across 133 Canadian secondary schools in British Columbia (n=14), Alberta (n=5), Ontario (n=51) and Quebec (n=63). 

COMPASS data are available upon reasonable request by completing a COMPASS Data Usage Application at: https://uwaterloo.ca/compass-system/information-researchers.

The University of Waterloo Office of Research Ethics Committee (ORE #30118) and participating school board ethics committees approved all procedures.

***Student-level measures***

Past 30-day (current) HTP use was assessed with a single question: “In the last 30 days, did you use any of the following? (Mark all that apply)” with one of the response options being “Heated tobacco product (a device that heats tobacco instead of burning it, such as IQOS or Heatstick).” Students who selected this response were categorized as current (past 30-day) HTP users, while those who did not were categorized as non-current HTP users.

Students also self-reported their gender (female, male, other, prefer not to answer); school grade (9, 10, 11, 12 or other, or secondary I, II, III, IV, V in Quebec), ethnicity (White, Black, Asian, Latin American/Hispanic, other, mixed); weekly spending money ($0, $1–5, $6–10, $11–20, $21–40, $41–100 or >$100); cigarette smoking behaviours (ever use and past 30-day use); and e-cigarette use behaviours (ever use and past 30-day use). Those who reported smoking in the past 30 days were considered current smokers; those who reported ever smoking but not in the past 30 days were considered ever smokers; and those who did not report ever smoking were considered never smokers. Similar definitions were used for e-cigarette use.

***School-level measures***

Consistent with other school-based studies,^18^,^19^ urbanicity was determined based on school postal codes and the classification of “rural” area and “small,” “medium” and “large urban” population centres by Statistics Canada.^20^ Based on this classification, we classified 12 schools as “rural,” 45 as “small urban,” 10 as “medium urban” and 53 as “large urban.”

***Proximity and density of retailers selling IQOS devices and HEETS***

Between November 2020 and March 2021, we used the IQOS search engine (https://ca.iqos.com/store/en/search) to identify retailers selling (1) IQOS devices and (2)HEETS (tobacco sticks used with IQOS) located within 6 km of each secondary school participating in the COMPASS study. 

We tracked each retailer’s name, address and estimated distance to the nearest school in our sample (if within 6 km of a school) on an Excel spreadsheet (Microsoft Corp., Redmond, WA, US). Using the postal codes of each school and each retailer, we geocoded each address and created circular buffer zones with 500m, 1000m and 1500m radius (0.31, 0.62 and 0.93 miles, respectively) from each school using geographic information system software ArcGIS (Esri, Redlands, CA, US). A 1000m radius circular buffer zone is believed to approximate how far students would actively commute, that is, walk or cycle, to school,^21^ and is consistent with previous literature examining the density and proximity of tobacco retailers and adolescent smoking.^14^ Given the lack of definite evidence in this area, we explored whether the association differed for closer (i.e. 500m) and farther (i.e. 1500m) distances. We used the number of retailers selling IQOS devices and HEETS within each circular buffer zone to identify the retailer proximity/density. The retail proximity/density data for each school were linked to student-level data for each school based on a unique school code.

***Analysis***

Descriptive statistics identified the mean number of retailers within 500m, 1000m and 1500m of each school. A null, multilevel regression model examined whether current HTP use varied across schools by calculating the intraclass correlation coefficient (ICC). 

The next set of multilevel models examined whether the presence of any retailers (i.e. proximity) selling (1) IQOS devices and (2) HEETS at each distance was associated with current HTP use in separate models (2 devices 3 distances = 6 models for proximity), while adjusting for province, school urbanicity, student-level characteristics (grade, gender, ethnicity, amount of spending money, cigarette smoking status, e-cigarette use status) and student-level clustering within schools. 

Another set of multilevel models examined whether an increasing number of retailers (i.e. density) selling (1) IQOS devices and (2) HEETS at each distance was associated with current HTP use in separate models (6 models total), while adjusting for the same covariates and student-level clustering within schools. 

We excluded data from 13 schools (3 in British Columbia, 1 in Alberta, 6 in Ontario and 3 in Quebec) that participated in the 2020–2021 COMPASS study but for which we did not have retailer data (n=5639 students). Students with missing outcomes (n=6811) or covariates (n=383) were excluded from the analyses (representing 15.0% of the sample), leaving a final sample of 40636 students. Students with missing outcomes tended to be male, other/mixed ethnicity and to not report their spending money; there were no significant differences in cigarette smoking or e-cigarette use status (data not shown). 

Descriptive statistics and regression models were completed using statistical software SAS, version 9.4 (SAS Institute Inc., Cary, NC, US).

## Results

In our sample, 0.80% of students reported using HTPs in the last 30 days (Table 1). While the prevalence of use was low across many demographic characteristics, students in Grade 12, those who identified their gender as other or preferred not to answer, and those of other/mixed ethnicity reported higher rates of HTP use. Similarly, current smokers and current vapers also reported higher rates of HTP use.

**Table 1:** Prevalence of current heated tobacco product use, overall and by demographic and behavioural characteristics, 2020–2021 COMPASS study (N = 40 636 students)

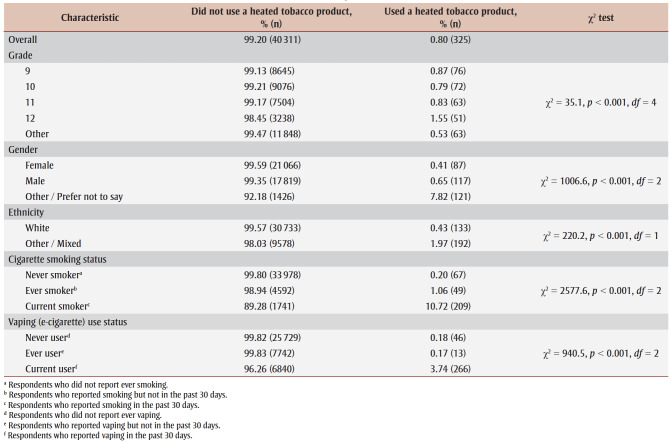

***Proximity of retailers selling IQOS devices and HEETS***

The overall percentage of schools in our sample that had at least one retailer within 500m, 1000m and 1500m of the school selling IQOS devices was 4.2%, 10.0% and 21.7%, respectively (Table 2). More schools had at least one retailer that sold HEETS within those distances (35.8%, 65.0% and 77.5%, respectively). Not surprisingly, there tended to be a higher proximity of retailers that sold IQOS devices and HEETS in large urban areas than in small or medium urban areas.

**Table 2:** Proximity and density of retailers selling IQOS devices and HEETS within 500 m, 1000 m and 1500 m of secondary schools, overall and by urbanicity, 2020–2021 COMPASS study (N = 120 secondary schools)

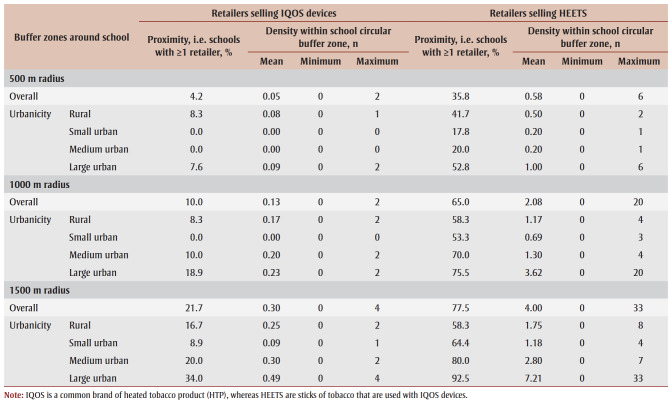

***Density of retailers selling IQOS devices and HEETS***

At 0.05, 0.13 and 0.30, respectively, the average density of retailers selling IQOS devices within 500m, 1000mand 1500m of each school in our sample was low (Table 2). In contrast, at 0.58, 2.08 and 4.00, respectively, the average density of retailers selling HEETS was much higher. As for the results for retailer proximity, there tended to be a higher density of retailers that sold IQOS devices and HEETS in large urban areas than in small or medium urban areas.

***Multilevel model results***

The null model suggests there is significant between-school variability in the likelihood of current HTP use among students [σ^2^_μ0_ = 0.326 (0.089); *p* < 0.001]; the school a student attended accounted for approximately 23.7% of the variability in the likelihood of currently using an HTP. The school-level prevalence of HTP use ranged from 0.02% to 2.90%, and 33schools had no students reporting HTP use (Table 2). 

After controlling for relevant covariates, the proximity to schools of retailers selling IQOS devices and HEETS was not significantly associated with current HTP use (Table 3). Similarly, after controlling for relevant covariates, the density of retailers selling IQOS devices and HEETS was not significantly associated with current HTP use (Table 4).

**Table 3:** Association between the presence of retailers selling IQOS devices and HEETS at various distances from a school and current use of heated tobacco products, 2020–2021 COMPASS study (N = 120 secondary schools)

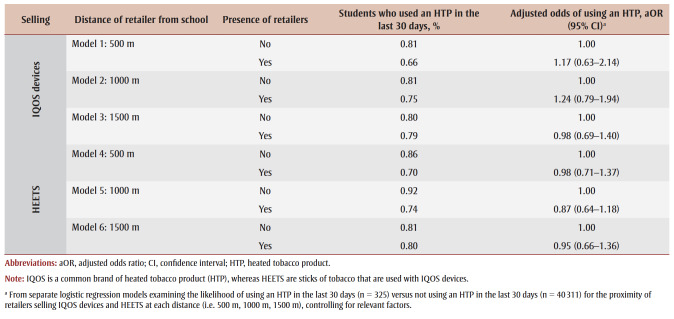

**Table 4:** Association between the density of retailers selling IQOS devices and HEETS and current use of heated tobacco products, 2020–2021 COMPASS study (N = 120 secondary schools)

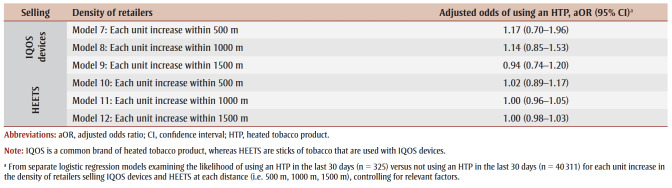

## Discussion

Our results indicate that less than 1% of students in our sample used HTPs at the time of the COMPASS survey in 2020–2021. While there was wide variability in the number of retailers selling IQOS devices and HEETS tobacco sticks near schools in our sample, and the school a student attended accounted for a significant amount of variability in HTP use, neither the proximity nor density of retailers selling IQOS devices were significantly associated with current HTP use. Similarly, neither the proximity nor density of retailers selling HEETS were significantly associated with current HTP use.

Consistent with previous studies,^9^,^10^,^12^ few students in our sample reported currently using HTPs. Students may be unaware of HTPs due to their relative novelty. HTP use is highest among current smokers compared to non-smokers and current vapers compared to non-vapers,^1^,^9^,^10^,^12^ suggesting that those who use tobacco and vaping products may be more inclined to use HTPs. Taking into account the diffusion of innovation theory, students who smoke and vape may be the first to adopt a new method of inhaling nicotine.^13^ Continued research and monitoring may help identify any rapid shifts in use by youth if HTP use gains momentum in Canada.

The prevalence of current HTP use varied widely across schools in our sample. HTPs are a relatively novel product with different levels of diffusion across areas (as illustrated by the differences in the number of retailers across population centres), which may contribute to the variability in use across schools. Since innovative technologies can diffuse and become more widespread in a population,^13^ and peers can influence tobacco use,^22^-^24^ the prevalence of HTP use may increase rapidly through use by a few influential students in a school. The school environment continues to be an important setting for tobacco prevention intervention, and it may be useful to target interventions to those schools at risk of experiencing a high prevalence of tobacco use.

Overall, there were more HEETS retailers than IQOS retailers: 72.5% of schools had a HEETS retailer within 1500m compared to 16.6% with an IQOS retailer, and schools had an average of 3.8 HEETS retailers within 1500m of the school compared to an average of 0.1 IQOS retailers. While the average number of retailers selling IQOS and HEETS within 1000m of each school was lower in our study than in a recent study conducted in Israel,^16^ the proportion of schools with at least one retailer selling IQOS devices or HEETS was similar. IQOS devices are typically sold in specialty stores such as IQOS boutiques and Q-labs, while HEETS products can be sold in non-specific stores that are more common around schools, such as convenience stores and gas stations. The lack of IQOS retailers identified near secondary schools in our sample suggests that students may find it more difficult to obtain IQOS devices than HEETS tobacco sticks, perhaps only leaving those students who already have an IQOS device to seek out HEETS products at retailers around their school. Adolescents could also be obtaining IQOS products through online sources like the IQOS website or through social sources (e.g. family members or friends). Additional research is needed to identify how students obtain IQOS devices in order to inform future retail policies or interventions.

This cross-sectional study did not identify a significant association between HTP retailer proximity and density and current HTP use by students, despite a high number of retailers close to schools. No existing government policies in Canada regulate the proximity or density of tobacco retailers around youth-friendly environments like schools. Given that students may notice tobacco marketing in the places they frequent, such as convenience stores and gas stations near schools, and this exposure may increase the likelihood that students use tobacco products,^14^,^15^ policy makers should consider zoning laws that limit the number of tobacco product retailers near schools.

***Strengths and limitations***

To our knowledge, this study is the first to examine the association between HTP retailer proximity and density on current HTP use by adolescents. The sample included a large, diverse sample of schools across four Canadian provinces. Limitations include the focus on IQOS products, the most widely available HTP brand in Canada. 

We only searched for retailers using the IQOS website and did not use search engines such as YellowPages or Google; we assumed that the IQOS website would have the most accurate listing of retailers selling their devices in order to direct potential customers to retailers. A study that compared a list of vape retailers obtained through online searches with that of a licensure database found that many confirmed vape stores identified through the online search were not on the licensure list.^25^ This suggests that online searches may provide a more comprehensive list of retailers relative to other document sources, possibly because they can be updated more regularly.^25^ We did not assess the accuracy of the search results either by visiting locations in person or calling retailers to confirm the products sold. Future studies could investigate the accuracy of the retailers provided by the website and whether this differs based on urbanicity. 

At the time of the search, IQOS devices were relatively new to the Canadian market. As the business expands, the IQOS retailer search engine updates the number of retailers selling IQOS devices and HEETS; therefore, the total numbers of HTP retailers surrounding schools may be underestimated. Continued monitoring is warranted to evaluate how changes in retailer proximity and density are associated with changes in student behaviours. 

Student data were based on self-report, which may be at risk of recall and social desirability bias; however, the use of passive-consent protocols limit self-selection and response bias that are common in studies of substance use behaviours.^26^

There was a high amount of missing outcome data. While there were some differences in the demographic characteristics of those with and without missing outcomes, there were no significant differences in cigarette smoking or e-cigarette use status. Given the large sample size for analysis, we believe there is sufficient statistical power to draw meaningful conclusions without data imputation.

## Conclusion

This was the first study to examine the association between HTP retailer proximity and density to schools and current use of HTPs by students. While the prevalence of current HTP use was low in our sample, the majority of schools had at least one retailer selling IQOS or HEETS within 1000m of the school and the school environment accounted for a high amount of variability in student HTP use. As there was no significant association between HTP retailer proximity/density and HTP use by students, students may be obtaining HTP products through other, non-retail sources including social sources. Additional monitoring of the distribution of HTP retailers and the prevalence of HTP use is warranted as knowledge, awareness and use of HTPs among youth may change.

## Acknowledgements

The authors would like to thank Q. Chen for geocoding and creating the final retailer dataset.

The COMPASS study has been supported by a bridge grant from the Canadian Institutes of Health Research (CIHR) Institute of Nutrition, Metabolism and Diabetes (INMD) through the “Obesity – Interventions to Prevent or Treat” priority funding awards (OOP-110788; awarded to SL), an operating grant from the CIHR Institute of Population and Public Health (IPPH) (MOP-114875; awarded to SL), a CIHR project grant (PJT-148562; awarded to SL), a CIHR bridge grant (PJT-149092; awarded to KP/SL), a CIHR project grant (PJT-159693; awarded to KP) and by a research funding arrangement with Health Canada (#1617-HQ-000012; contract awarded to SL), a CIHR-Canadian Centre on Substance Use and Addiction (CCSA) team grant (OF7 B1-PCPEGT 410-10-9633; awarded to SL), and a project grant from the CIHR Institute of Population and Public Health (IPPH) (PJT-180262; awarded to SL and KP).

A SickKids Foundation New Investigator Grant, in partnership with CIHR Institute of Human Development, Child and Youth Health (IHDCYH) (Grant No. NI21-1193; awarded to KAP) funds a mixed methods study examining the impact of the COVID-19 pandemic on youth mental health, leveraging COMPASS study data. The COMPASS-Qubec project also benefits from funding from the Ministre de la Sant et des services sociaux of the province of Quebec and the Direction rgionale de sant publique du Centre intgr universitaire de sant et de services (CIUSSS) de la Capitale-Nationale.

The funding sources had no role in the study design; in the collection, analysis and interpretation of data; in the writing of the manuscript; or in the decision to submit the article for publication.

## Conflicts of interest

Scott Leatherdale is one of this journal’s associate scientific editors, but has recused himself from the review process for this article.

The authors have no other conflicts of interest to declare.

## Authors’ contributions and statement

HM – Conceptualization, data curation, formal analysis, writing – original draft, writing – review & editing.

STL – Data curation, funding acquisition, writing – review and editing, conceptualization, investigation, methodology, project administration, resources. 

AGC – Conceptualization, formal analysis, methodology, supervision, writing – review& editing.

All authors approved the final manuscript as submitted and agree to be accountable for all aspects of the work.

The content and views expressed in this article are those of the authors and do not necessarily reflect those of the Government of Canada.
